# Characteristics of Acute Appendicitis before and during the COVID-19 Pandemic: Single Center Experience

**DOI:** 10.1155/2022/4541748

**Published:** 2022-02-24

**Authors:** A. Bosak Veršić, M. Šestan, I. Ćepić, H. Nikolić, N. Bukvić, S. Sršen Medančić, D. Hasandić, M. Zelić

**Affiliations:** ^1^Pediatric Surgery Department, University Hospital Rijeka, Rijeka 51000, Croatia; ^2^Medical Faculty, University Hospital Rijeka, Rijeka 51000, Croatia; ^3^Surgery Department, University Hospital Rijeka, Rijeka 5100, Croatia

## Abstract

The aim of the study was to investigate whether the COVID-19 pandemic caused an increased incidence of complicated appendicitis due to the late presentation when compared to the pre-COVID-19 period. *Summary Background Data*. Acute appendicitis is one of the most common surgical emergencies. During the coronavirus-19 (COVID-19) pandemic, there has been a reported delay in the presentation of some urgencies to the emergency hospital departments. *Methods*. A total of 427 patients who underwent surgical treatment due to suspected acute appendicitis from June 2019 to November 2020 were retrospectively included in this study. The patients were divided into two groups: the first (pre-COVID-19) group consisted of patients who had surgery before the onset of COVID-19 pandemic (*n* = 240), while the second (COVID-19) group consisted of those who were operated during the COVID-19 pandemic (*n* = 187). The primary outcome of the study was to compare the incidence of perforated appendicitis before and during the onset of COVID-19. *Results*. Overall, 84 patients (19.67%) were diagnosed with perforated appendicitis. We found a weak significance (*p*=0.085) in the rate of perforated appendicitis between the pre-COVID-19 (17.08%) and the COVID-19 era (22.99%). *Conclusions*. We did not observe any significant difference in the complications of acute appendicitis before and during the COVID-19 pandemic in a university hospital in Rijeka. An emergent medical care should always be accessible.

## 1. Introduction

At the end of 2019, a new coronavirus was identified as the cause of a cluster of pneumonia cases in Wuhan, China. It rapidly spreads, resulting in an epidemic throughout China, followed by an increase in the number of cases in countries around the world. In February 2020, the World Health Organization (WHO) designated the disease COVID-19, which stands for coronavirus disease 2019 [1, 2]. Many countries struggled to stave off the rapid spread of COVID-19 by implementing different strategies. In most countries, including Croatia, authorities have instructed the public to stay at home and to avoid unnecessary socializing. In our hospital, a significant decrease in the number of emergency department (ED) examinations has been noted. However, urgent medical cases have continued to appear along the current outbreak, and their diagnosis needs to be made appropriately. Delayed diagnosis and treatment of these conditions may lead to a significant morbidity that may outweigh the harm caused by COVID-19 infection [3]. Acute appendicitis is one of the most common abdominal surgical emergencies in general with a 7% lifetime risk [4–7]. Eventhough antibiotics have been described as a type of therapy for uncomplicated appendicitis, surgery still remains the preferable treatment modality [8]. Early diagnosis of appendicitis and consequent appropriate surgical treatment is important as it may prevent complications such as appendix perforation, abscess formation, and other postoperative complications including death [9]. During the COVID-19 pandemic, it is expected that more patients will seek out medical care in the later stage of the disease along with significant complications [2, 10]. The aim of this study is to investigate the characteristics and management of patients with acute appendicitis in our hospital during the first wave of COVID-19 pandemic compared to the pre-COVID-19 period.

## 2. Patients and Methods

**Figure 1:**
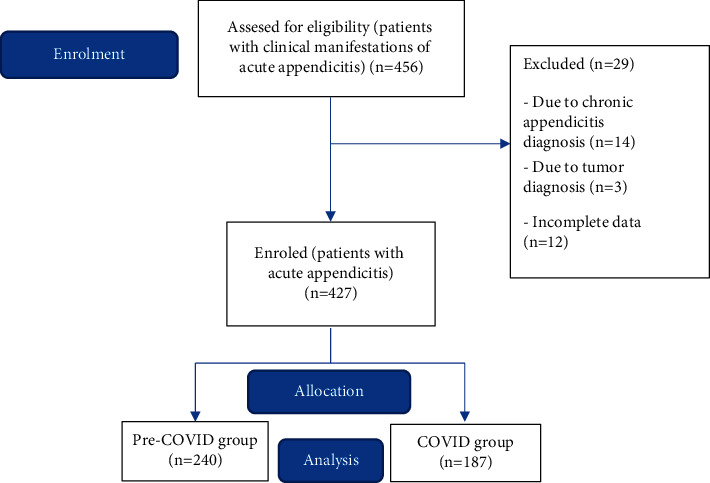
Flowchart of patient inclusion. A total of 456 patients were included in the study, 240 in the pre-COVID-19 group and 187 in the COVID-19 group.

We performed a retrospective monocentre observational study that included adult and pediatric patients surgically treated due to suspected acute appendicitis in the period from 1st of June 2019 to 31st of December 2020. The patients were divided into two groups: those treated in the pre-COVID-19 era and the ones treated during the COVID-19 era. We included all patients 2–86 years old who were referred to our surgical teams in a university hospital in Rijeka, Croatia, with suspected appendicitis. Initially, a total of 456 patients were included in the study (Figure 1). The data were collected from the hospital database. 14 patients who underwent interval appendectomy (due to chronic appendicitis) were excluded from the study. Additionally, 3 patients who were diagnosed with appendicular malignancies were also excluded from the study, as well as 12 patients with insufficient data available. Approval was obtained from the ethics committee at the hospital. In regard to treatment, patients were included if they were treated surgically with laparoscopic or open appendectomy. Data extracted from patients' notes included demographic and clinicopathological variables, duration of symptoms upon presentation in the ED, and postoperative length of stay. Intraoperative findings were recorded and classified as acute appendicitis if gross acute inflammation of the appendix was seen. We divided the diagnosis into 3 groups: normal appendicitis, uncomplicated appendicitis, and perforated appendicitis. The primary outcome of the study was to compare the incidence of perforated appendicitis before and during the onset of COVID-19. In order to assess these outcomes, the cohort was allocated into two groups. In the first group, pre-COVID-19 patients were hospitalized in the period from 1^st^ of June 2019 to 10^th^ of March 2020. In the second group, COVID-19 era patients were hospitalized in the period from 11^th^ of March 2020 to 31^st^ of December 2020.

### 2.1. Statistical Analysis

Statistical analyses were performed using MedCalc for Windows, version 19.4 (MedCalc Software, Ostend, Belgium). Patient cohorts were compared using chi-square, Student's *t*-test, and Mann–Whitney *U* tests where appropriate. A *p* value of <0.05 was considered significant.

## 3. Results

Within the two periods of this study, a total of 427 patients were included, 240 in the pre-COVID-19 era and 187 in the COVID-19 era. We noted a 22% drop in the number of appendicitis patients treated in the COVID-19 era when compared to the pre-COVID-19 period. Baseline demographic data of all patients are given in Table 1. There were 104 pediatric and 323 adult patients treated. The median age of the entire population treated was 31 years. Male patients predominated (53.63%). There were no significant demographic differences across time periods.

Clinical characteristics are given in Table 2.

Median length of duration of symptoms upon presentation was 1 day with no difference between the two groups observed. There was no significant difference in the surgical treatment modality (open vs. laparoscopic) between the two periods (*p*=0.368). Overall, 84 patients (19.67%) were diagnosed with perforated appendicitis. In the COVID-19 era, there was an incidence of 22.99% of perforated appendicitis and 17.08% in the pre-COVID-19 era (*p*=0.085) which is weakly significant. Our negative appendectomy rate overall and in pre-COVID-19 and COVID-19 era was 11.24%, 13.75%, and 8.02%, respectively. Duration of symptoms and length of hospitalization were longer in patients with perforated appendicitis (overall). The average duration of symptoms in all patients was 24 hours; however, when observing perforated appendicitis only, it was 48 hours. Radiologic investigations (US and CT) are used more frequently in patients with more severe clinical presentation (*p*=0.006, *p* < 0.001). We did not find any increase in radiologic investigations between the pre-COVID-19 and COVID-19 era.

The duration of hospitalization on average was 5 days, and in those with perforation, it was 7 days. We did not find a significant difference in the duration of symptoms (*p*=0.065) and in duration of hospitalization (*p*=0.990) before the COVID-19 and during the COVID-19 era. The duration of hospitalization, in perforated appendicitis only, was shorter in the COVID-19 era (*p*=0.025). We noticed that there was a significantly higher percentage of antibiotics being used when observing appendicitis patients in the COVID-19 era (*p* < 0.001) as well as when observing nonappendicitis cases in the same period. We did not find any increase in radiologic investigations between the pre-COVID-19 and COVID-19 era.

## 4. Discussion

According to our knowledge, this is the only study analyzing appendicitis management during the COVID-19 pandemic in Croatia.

The main results of our study showed a weakly significant higher rate of complicated appendicitis during the COVID-19 era when compared to the pre-COVID-19 period. There were not any demographic differences between the time periods. The time between the onset of symptoms and presentation at the emergency hospital department did not prolong in the COVID-19 era. There was no change in a surgical management modality and length of hospital stay in the two time periods observed.

During the COVID-19 pandemic, most of the medical centers worldwide postponed or canceled regular elective procedures. The same was in Croatia. Surgical procedures were limited to urgent surgical, oncology, or trauma cases. The aim was to minimize unnecessary work load in the healthcare system and reduce emergency department patient encounters [11–13]. Furthermore, a significant increase in delayed medical treatment for various emergencies during the COVID-19 pandemic period has been noted [13, 14]. Several recent studies of acute appendicitis during COVID-19 pandemic clearly show that staying at home due to public health safety orders negatively impacted children and adults who developed appendicitis [6, 10, 13–15]. During the COVID-19 pandemic, an increased rate of perforated appendicitis, compared to the pre-COVID-19 period, was reported in several published studies [6, 10, 14, 16–18]. Moreover, it has been noted that patients with perforated appendicitis have had an increased rate of complications and length of hospital stay [3, 11, 12, 17, 19, 20]. COVID-19 is characterized by an unpredictable disease course, ranging from asymptomatic infections to severe and life-threatening situations. Pan et al. observed that 50.5% of patients infected with COVID-19 reported gastrointestinal symptoms along with the presence of respiratory symptoms [21]. Therefore, the diagnosis of acute appendicitis may be challenging during the COVID-19 pandemic. Appendectomy is the gold standard of treatment for acute appendicitis. However, recent studies suggest that conservative management with intravenous antibiotics can be used as an alternative [8, 22]. On the other hand, appendectomy remains the most effective and safe treatment option and provides curative treatment without recurrence risk. In this study, we have shown that in our hospital, the number of patients presented to the ED with clinical signs of acute appendicitis decreased during the time when the number of COVID-19 cases started to increase. It is possible that patients choose not to present to the ED. The decision might have been influenced by the aggressive national policy of social isolation in Croatia. Furthermore, there might have been an element of patient's concern about patient-to-patient SARS-CoV-19 airborne transmission in the ED.

There was no significant difference between the two observed groups in terms of length of preoperative symptoms or type of surgery, need for postoperative peritoneal drainage, or the distribution of complicated versus uncomplicated appendicitis.

There are several possibilities that could explain this trend. This could be backed-up by some reports describing a successful resolution of mild appendicitis treated symptomatically by patients at their own private home settings [23, 24]. Additionally, some patients might have been treated by primary healthcare practitioners. The reason for no significant increase in complicated appendicitis might also lie in the fact that the treatment in our hospital is covered by national health insurance and access was never limited, denied, or postponed by COVID-19 testing.

Similar to our results, there are studies done in Italy, Turkey, Israel, and Germany. Although the fear of the COVID-19 pandemic resulted in a delayed diagnosis of some serious pediatric and adult diseases, an increase in the prevalence and severity of acute appendicitis was not demonstrated even in most COVID-19 affected areas in Italy [11, 25]. German authors also noted an overall drop in the number of uncomplicated appendicitis cases and no change in the rates of perforated appendicitis [24]. A study from Israeli tertiary center reported the same incidence of perforated appendicitis before and after the onset of the pandemic [26].

Similarly, no increase in the diagnosis of perforated appendicitis during the pandemic period was obtained by Turkish authors [27].

Some studies, on the contrary, have reported different results. In Japan, the authors found a delay in presentation for acute appendicitis with a higher incidence of perforation [28]. Higher rates of perforated appendicitis were also described by several American authors [15, 29], as well as in the study from a poor area in Nepal [30].

Our negative appendectomy rates observed correlate with those described in the literature [7].

The common conclusion of all authors reporting a higher incidence of complicated appendicitis was disruption of continuous medical care due to public healthcare crisis.

We think that the reason for our results was that the system was as accessible during the pandemic as it was before the period, and there were fewer “nonurgent” cases going throughout emergency departments. In addition, at the time, our system was not preoccupied with COVID-19 patients.

The main limitations of the study are the retrospective character of the study and the fact that it is a single centre experience. However, since the pandemic still continues, an expanded study that would include all main centers in the country will be considered. Furthermore, we limited the period of the study to 9 months pre and post-COVID, since at the time, it was not possible to predict the duration of the situation.

## 5. Conclusion

We did not observe any significant difference in the rate of complications of acute appendicitis before and during the COVID-19 pandemic in a university hospital in Rijeka. We think we obtained this result because, despite the appeals not to go to the hospital if not necessary, emergency healthcare still remained as accessible as before the COVID-19 pandemic. Additionally, the reason might be that the pandemic did not affect our region at the time as severely as it did some other countries.

## Figures and Tables

**Table 1:** Comparison of demographic data between the two groups.

Variable	Total *N* = 427	Pre-COVID group *N* = 240	COVID group *N* = 187	*P* value
Age, median (range)	31 (2–92)	29 (2–86)	33 (4–92)	0.061
Sex, *n* (%)				
Male	229 (53.63)	126 (52.50)	103 (55.08)	0.596
** **Female	198 (46.37)	114 (47.50)	84 (44.92)	0.596

There was no difference in the demographic characteristics between the two groups.

**Table 2:** Treatment characteristics of the patients in the two groups observed.

Variable	Total *N* = 427	Pre-COVID group *N* = 240	COVID group *N* = 187	*P* value
Duration of symptoms, hours, self-reported	24 (1–240)	24 (3–168)	24 (1–240)	0.065

Treatment modality, *n* (%)				
** **Open appendectomy	104 (24.36)	54 (22.50)	50 (26.74)	0.368
** **Laparoscopic appendectomy	323 (75.64)	186 (77.50)	137 (73.26)	

Administration of antibiotics, *n* (%)	332 (77.75)	160 (66.67)	172 (91.98)	<0.001

Grade of appendicitis, *n* (%)				
** **Normal appendix	48 (11.24)	33 (13.75)	15 (8.02)	0.085
** **Uncomplicated appendicitis	295 (69.09)	166 (69.17)	129 (68.98)	0.085
** **Complicated appendicitis	84 (19.67)	41 (17.08)	43 (22.99)	0.085

Postoperative peritoneal drain, *n* (%)	152 (35.60)	78 (32.50)	74 (39.57)	0.130
Length of admission (days)	5 (2–33)	5 (2–33)	5 (3–32)	0.990

Final diagnostic imaging, *n* (%)				
** **Ultrasound	137 (32.08)	80 (33.33)	57 (30.48)	0.531
** **CT	39 (9.13)	24 (10.00)	15 (8.02)	0.481

We did not notice any significant differences in the duration of symptoms between the pre-COVID-19 and COVID-19 groups. There was no change in the representation of operational techniques. We noticed higher rate of antibiotics administration during the COVID-19 period. The incidence of complicated appendicitis was not higher in the COVID-19 period. Also, the length of hospital stay did not change during the COVID-19 era. Imaging techniques were equally represented before and in the COVID-19 era.

## Data Availability

The data used to support the findings of this study are available from the corresponding author upon request.

## Supplementary Materials

Supplementary Materials Strobe checklist: checklist of the items in this cohort study.
